# Treatment of splenic marginal zone lymphoma of the CNS with high-dose therapy and allogeneic stem cell transplantation

**DOI:** 10.1186/2162-3619-1-32

**Published:** 2012-10-16

**Authors:** Christoph Busemann, Andrej Gudzuhn, Carsten Hirt, Michael Kirsch, Silke Vogelgesang, Christian A Schmidt, Gottfried Dölken, William H Krüger

**Affiliations:** 1 Internal Medicine C (Hematology and Oncology, Palliative Care, Transplant Centre), University Hospital Greifswald, Ernst-Moritz-Arndt-University, Greifswald, Germany; 2 Institute for Radiology, University Hospital Greifswald, Ernst-Moritz-Arndt-University, Greifswald, Germany; 3 Department of Neuropathology, Institute for Pathology, University Hospital Greifswald, Ernst-Moritz-Arndt-University, Greifswald, Germany; 4 Medizinische Klinik C (Hämatologie und Onkologie, Transplantationszentrum), Ernst-Moritz-Arndt-Universität Greifswald, Ferdinand-Sauerbruch-Straße, Greifswald, 17475, Germany

**Keywords:** Non-Hodgkin’s lymphoma, Allogeneic stem cell transplantation, Graft-versus-lymphoma effect, Marginal zone lymphoma

## Abstract

Therapy of indolent lymphomas with involvement of the central nervous system (CNS) has not been standardized so far. A 42-year old male patient presented with neurological signs because of leukemic splenic marginal zone lymphoma (SMZL) manifested in bone marrow, lymph nodes and CNS. Due to the aggressiveness of the disease and the young age of the patient, an intensive immunochemotherapy followed by high-dose therapy with busulfan, thiotepa and fludarabine and subsequent unrelated allogeneic stem cell transplantation (alloSCT) was performed. The haemopoietic stem cells engrafted in time and the patient is doing well (ECOG 0) without evidence for active lymphoma three years after transplantation. Highly sensitive tests by specific quantitative real-time polymerase chain reaction for presence of lymphoma cells in blood and bone marrow indicated also a molecular remission. The reported case shows the feasibility of high-dose therapy and allogeneic stem cell transplantation in high-risk patients with CNS-involvement of indolent non-Hodgkin’s lymphoma. In addition, the case supports the hypothesis that the graft-versus lymphoma effect after alloSCT is also active within the CNS.

## Background

Splenic marginal zone lymphoma (sMZL) has usually a very indolent course and affects the elderly people. Therapy consists of splenectomy and mild chemotherapy upon clinical course [1-5]. Secondary affection of the central nervous system by non-Hodgkin’s lymphoma is associated with a poor prognosis [6]. Here we present the uncommon case of a young man suffering from a very aggressive sMZL with primary affection of the CNS and the treatment with allogeneic stem cell transplantation for consolidation.

## Case presentation

**Figure 1:**
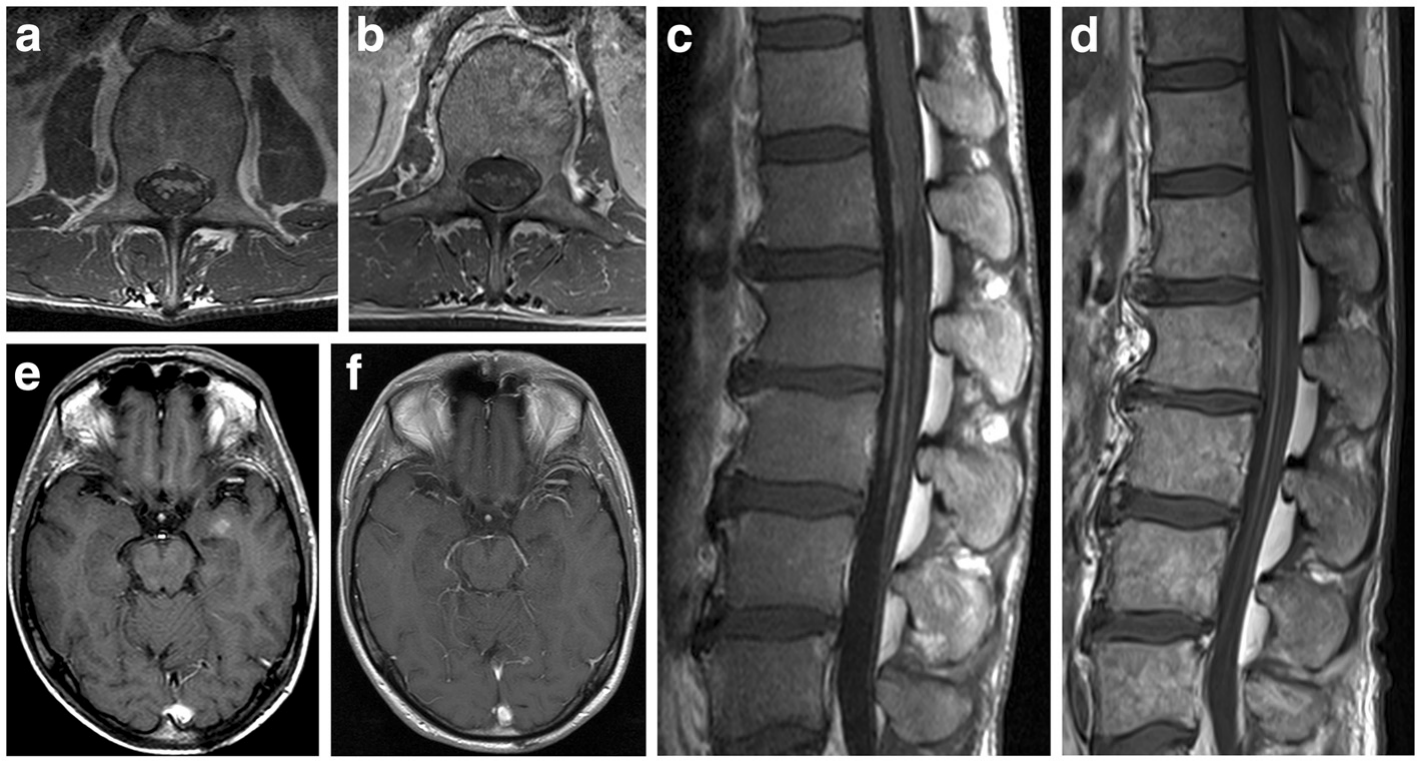
**MR images of LWS (a-d) and cranium (e-f) at initial diagnosis (a, c, e) and at 24 months after allogeneic transplantation (b, d, f).** The size and the intensity of contrast-agent enhanced structures within CNS have regressed after stem cell transplantation.

A 42-year old male presented with paraplegia, reduced vibration sense and neuropathic pain in the legs aggravating over the last six months. The further medical history was empty. Clinical examination and computed tomography revealed generalized lymphadenopathy and splenomegaly. B-symptoms were absent. Magnetic resonance imaging (MRI) of the CNS detected a signal intense 9 mm sized lesion within the temporal lobe and thickened filaments of the cauda equina with contrast medium enhancement, suggestive for a malignant meningeosis (Figure 1). The LDH has been elevated in serum (284 U/l, upper normal value: 253 U/l). Electrophoresis and immunofixation gave no hint for a monoclonal gammopathy.

**Figure 2:**
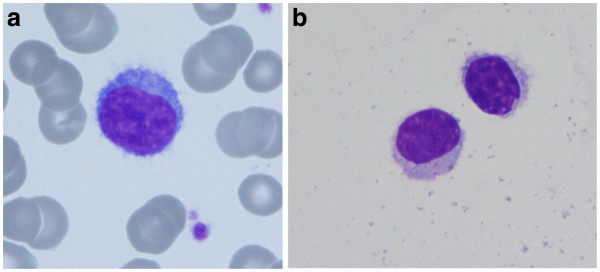
Villous lymphocytes in the peripheral blood (a) and in the cerebrospinal fluid (b).

**Figure 3:**
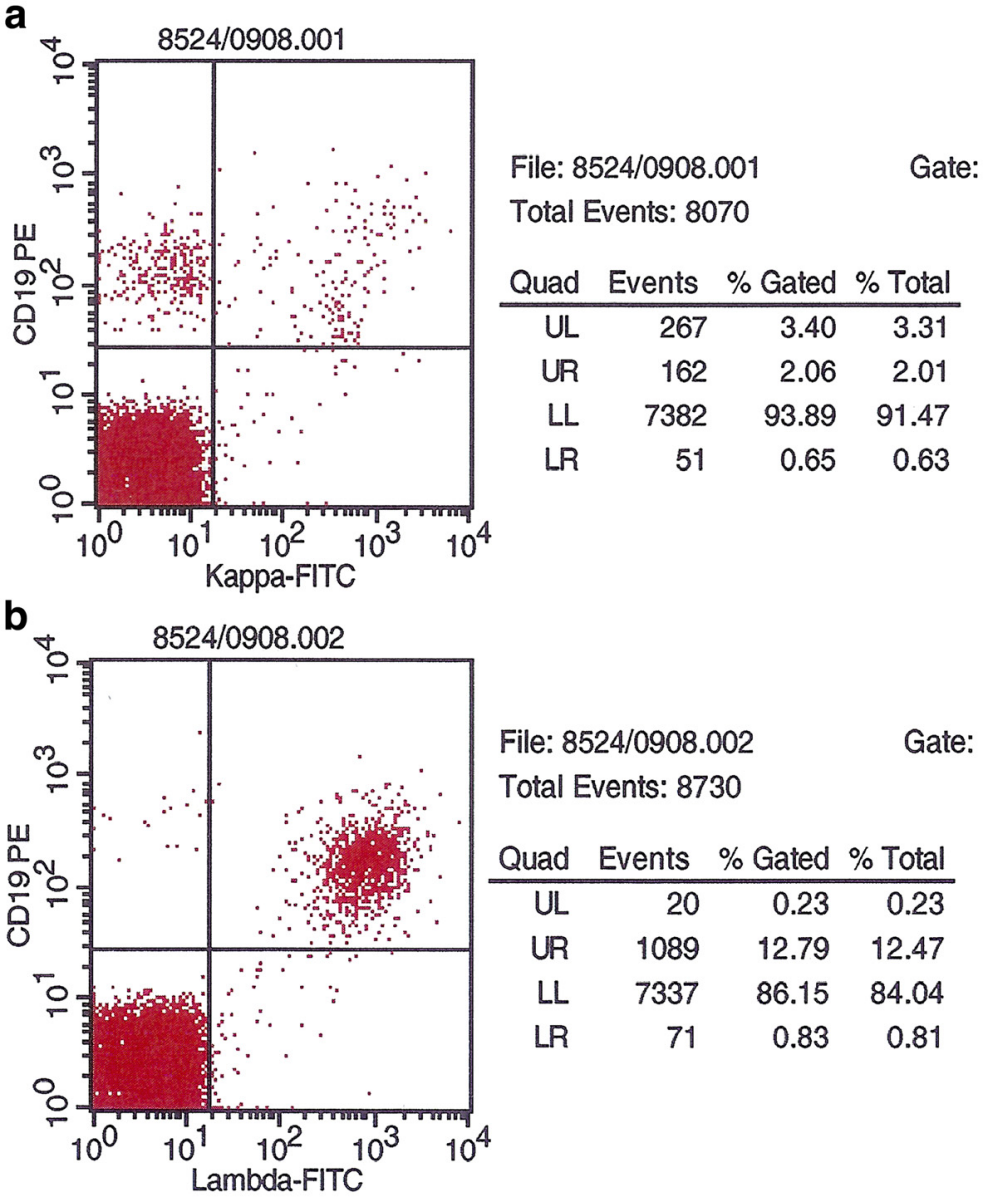
a: Kappa (negative) and b: Lambda light (positive, right upper quadrant) chain expression by MZL-cells from cerebrospinal fluid, FACS-analysis prior to therapy.

**Figure 4:**
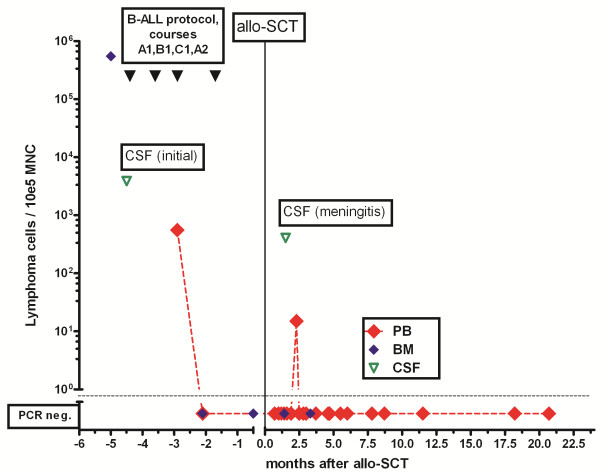
**Detection of lymphoma cells in bone marrow, blood and CSF by specific rtPCR.** BM: bone marrow, PB: peripheral blood, CSF: cerebro-spinal fluid.

Peripheral blood showed a lymphocytosis of 34,000/μl with predominance (92.2% in differential count) of small, mature and partially villous lymphocytes (Figure 2). The immunophenotype was compatible with SMZL (CD19^+^, CD20^+^, cy79a^+^, expression of sIgM, FMC7 and lambda light chain and partial expression of CD5 and CD38, but not CD10, CD11c, CD23, CD103 and CD138). Diagnosis of splenic marginal zone lymphoma was confirmed by histological examination of a lymph node. In the cerebrospinal fluid (CSF) lymphoma cells were detected by morphology (Figure 2) and flow cytometry (Figure 3). Cytogenetics showed an additional isochromosome 3q and a translocation t(2;7)(p11;q21-22) in line with the diagnosis of splenic marginal zone lymphoma. The IgH rearrangement of lymphoma cells was sequenced to establish a specific quantitative real time polymerase chain reaction (PCR) for detection of minimal residual disease (MRD) (Figure 4). PCR-analysis confirmed malignant lymphomatosis in CSF.

Therapy was initiated combining immunochemotherapy including rituximab, high dose methotrexate (MTX) and cytarabine (Ara-C) plus intrathecal application of MTX, Ara-C and dexamethasone according the B-ALL protocol [7]. With exception of persistent slight enlargement of the spleen, the lymphoma showed a complete response, including the neurological symptoms.

Allogeneic stem cell transplantation (alloSCT) was scheduled for consolidation since the lymphoma showed an aggressive behavior in a young patient. The experimental nature of this approach and conventional alternatives were discussed comprehensively with the patient and his wife. It is the only therapy offering the change of curing. Additionally, the aggressive course and the uncommon manifestation are suggestive for an early transformation of lymphoma. After conditioning with i.v.-Busulfan 6.4 mg/kg bodyweight, Thiotepa 10 mg/kg, Fludarabin 150 mg/m^2^ and ATG 6 mg/kg the patient was allografted with 10.2 x 10^6^ CD34^+^ cells per kg bodyweight from a matched unrelated donor. Cyclosporine-A and short-course MTX were given for GvHD-prophylaxis. Supportive therapy followed standard conditions and grade I renal toxicity was noted per the Bearman criteria [8]. Granulocyte engraftment (1,000 cells/μl) occurred at day +17 and platelets exceeded 20,000/μl at day +26, independently from transfusion. The patient developed a cvl-related septicaemia with Enterococcus faecalis and bacterial meningitis with Gram-negative rods. Both complications resolved under adequate antibiosis. Donor chimerism in the bone marrow was 100% at day +43 and the patients was discharged.

Signs of an acute or chronic graft-versus-host disease were not seen. Control MRIs from the central nervous systems showed continuing complete regression of the lymphoma (Figure 1f) Three years after allogeneic stem cell transplantation the patient is doing well (ECOG 0) without any neurological abnormalities. Molecular follow-up of minimal residual disease in blood or marrow by rtPCR revealed negative results after SCT (Figure 4). The patient is in sustaining complete remission according to hematologic, radiologic and molecular criteria.

## Conclusions

Extranodal manifestation of low grade non-Hodgkin lymhomas in the central nervous system is extremely rare. The synopsis of all diagnostic findings in the presented case resulted in the diagnosis of a splenic marginal zone lymphoma with meningeal lymphomatosis, infiltration of the temporal lobe and infiltration of the cauda equina despite the unavailability of a spleen histology as the diagnostic hallmark of this disease. The morphological features of the peripheral blood cells have been already very suggestive of SMZL and the differential diagnosis of variant form of hairy cell leukemia was excluded by immunophenotyping. Partial expression of CD5 as observed in this patient is found in approximately 20% of SMZL cells, mantle cell lymphoma is not plausible because of morphology and negativity for t(11;14) in FISH analysis. The translocation t(2;7)(p11;q21-22) is previously characterized in a few cases of SMZL and in CD5-negative MBL [9,10]. This translocation seems to link CDK6 to the IG kappa locus [11]. Trisomy 3 is a current but not specific chromosomal aberration which is found in a variety of B-cell lymphomas [9]. Summarized, the results of all analyses confirmed the extraordinary diagnosis of SMZL with involvement of the central nervous system.

Only three cases with CNS manifestation of a SMZL have been described in the literature so far. All of them suffered from meningeosis, one patient had additionally a cerebral manifestation and another patient an infiltration of the optic nerve. The treatment results have been different, two patients had good responses to chemotherapy and a third one had a refractory disease [12-14].

Normally, splenic marginal zone lymphomas have a favorable prognosis. Conventional therapy (splenectomy, purine analogs or rituximab, antiviral therapy in concomitant HCV infection) is not curative, but can lead to long lasting remissions. Adverse prognostic factors have been identified. Chacón et al. found extranodal site involvement (apart from liver and spleen) after splenectomy reducing median overall survival to 52 months, while overall survival in patients without extranodal site involvement was not reached at 113 months [15]. Elevation of LDH at initial diagnosis is discussed as adverse prognostic factor in a published scoring system [16]. Aggressive treatment forms like high-dose therapy (HDT) followed by autologous stem cell transplantation (auto-SCT) is a valuable option for younger patients with high-grade NHL of central nervous system [17]. This therapeutical approach has no plausible curative potential in case of indolent lymphomas, however.

Due to the special features indicating the aggressiveness of the disease and the young age of our patient, we recommended an intensive proceeding including therapy with drugs penetrating excellently into the CSF (MTX, Busulfan, Thiotepa, Fludarabine) followed by allogeneic stem cell transplantation from an unrelated donor in order to cure from lymphoma. SMZL disappeared completely, even on the molecular level (Figure 4, Figure 1).

Graft-versus-leukemia/lymphoma-effects are essential for the eradication of residual hematological malignancies after allogeneic BSCT [18], but the information about GvL-acitivity within the CNS is still limited. A case of a PCNSL treated with allogeneic peripheral BSCT has been described: the manifestation of acute GvHD could be correlated with the disappearance of intracerebral lymphoma [19]. Another group described donor-derived lymphocytes in the cerebrospinal fluid after transplantation [20]. Furthermore, the successful treatment by allogeneic BSCT of patients with isolated CNS relapse of acute leukemia has been published [21]. In a prior publication we reported the successful therapy of CNS-relapse of high-grade NHL with alloSCT, immunomodulation and irradiation [22]. The clinical and molecular remission of SMZL in this case strongly suggest contribution of GvL-effects, however, the relative short follow-up of three years allows no definite conclusion.

The actual case in conjunction with preceding publications should encourage the physicians involved into therapy of lymphomas of the CNS to consider allogeneic stem cell transplantation as a valuable salvage therapy and as a possible primary therapy in high-risk patients. Investigation of this approach in clinical trials and further research about the activity of donor cells in the CNS after alloSCT are mandatory.

### Consent statement

Informed written consent was obtained from the patient for allogeneic stem cell transplantation and for scientific evaluation of the transplantation and the results.

## Competing interests

No author has any competing interests.

## Authors’ contributions

Data interpretation and manuscript writing: CB, AG, CAS, WHK Cytopathology and flow cytometry: CB, AG Histopathology: SV Molecular analysis: CH Radiological diagnostics: MK Treatment of the patient: CB, AG, CAS, GD, WHK. All authors read and approved the final manuscript.

## Statement of prior presentation

This case report has not been previously published.
